# Evaluation of the Efficacy and Tolerability of Bedaquiline in Drug-Resistant Tuberculosis

**DOI:** 10.7759/cureus.115620

**Published:** 2026-09-01

**Authors:** Hitesha H Trivedi, Suraj Tripathi, Parth Patel, Mittal C Balat, Mohmed Sohel Shaikh, Nilesh Patil

**Affiliations:** 1 Department of Pharmacology and Therapeutics, Zydus Medical College and Hospital, Dahod, IND; 2 Department of Pharmacology, Zydus Medical College and Hospital, Dahod, IND; 3 Department of Respiratory Medicine, Zydus Medical College and Hospital, Dahod, IND

**Keywords:** antitubercular therapy, bedaquiline, drug-resistant tuberculosis, sputum conversion, treatment tolerability

## Abstract

Introduction: Multidrug-resistant tuberculosis (TB) poses a serious threat to public health globally because of limited treatment options and high levels of antimicrobial resistance. Bedaquiline is an important component of contemporary multidrug treatment regimens for drug-resistant TB. This study aimed to evaluate treatment outcomes and tolerability among patients receiving a bedaquiline-containing regimen, including sputum conversion, laboratory parameters, adverse drug reactions (ADRs), and treatment compliance. Materials and methods: The study was a hospital-based prospective observational study conducted among 64 patients with drug-resistant TB who attended the drug-resistant TB clinic at Zydus Medical College and Hospital, Dahod, from April 2024 to September 2025. Patients on a bedaquiline-based regimen were followed up monthly to assess sputum conversion, laboratory tests, ADRs, and treatment compliance. Results: Among 64 patients with drug-resistant TB, sputum conversion was achieved in 61 (95.3%), with no significant association between conversion and baseline demographic or clinical characteristics (all p > 0.05). Universal rifampicin resistance (100%) and high levels of isoniazid resistance were observed, whereas susceptibility was observed in 98.4% of isolates to injectable anti-TB drugs and in 64.1% to moxifloxacin. These in vitro drug susceptibility findings should not be interpreted as evidence of comparative clinical efficacy. Follow-up demonstrated preserved renal function, with mild but significant changes in liver function and electrolytes, whereas nausea (13, 20.3%) and electrolyte abnormalities (15, 23.4%) were the most frequent ADRs. Conclusions: A high sputum conversion rate of 95.3% (61/64) was observed during follow-up among patients receiving a bedaquiline-containing multidrug regimen. Most reported adverse events were mild; however, biochemical changes and QT prolongation were observed and require continued clinical monitoring. Given the prospective observational design, the absence of a comparator group, the concomitant use of multiple anti-TB drugs, and the limited follow-up duration, the independent contribution of bedaquiline to the observed treatment outcomes, as well as its long-term efficacy and safety, cannot be determined from the present study.

## Introduction

About one-third of the world’s population is estimated to be infected with tuberculosis (TB). TB is a major cause of infectious disease-related mortality worldwide. India accounts for about 32% of an estimated 390,000 multidrug-resistant or rifampicin-resistant TB (MDR/RR-TB) cases worldwide in 2024, considerably higher than other countries such as China, the Philippines, and Russia, which together account for half of the global MDR/RR-TB incidence. According to the World Health Organization (WHO) Global Tuberculosis Report 2025, an estimated 10.7 million people developed TB globally in 2024, while approximately 1.23 million people died from the disease, including 150,000 deaths among people living with HIV [1]. Recently, the development of drug resistance in TB has been attributed to various factors, which include inappropriate prescribing of TB medications by healthcare professionals, the availability of substandard drugs, and poor treatment adherence, leading to treatment interruption [2]. Monoresistant TB refers to TB caused by *Mycobacterium tuberculosis* that is resistant to only one first-line anti-TB drug, while remaining susceptible to the other first-line agents. Common examples include isoniazid-resistant TB or RR-TB. Its treatment differs from drug-susceptible TB and should be guided by drug-susceptibility testing. Multidrug-resistant TB (MDR-TB) is a clinical form of TB caused by *Mycobacterium tuberculosis* strains that exhibit resistance to at least isoniazid and rifampicin, the two most effective first-line anti-TB agents. MDR-TB represents a major therapeutic challenge because resistance to these cornerstone drugs substantially limits treatment options and reduces treatment success rates. Isoniazid and rifampicin are recognized as the most effective first-line drugs for the treatment of TB [3].

Patients with MDR/RR-TB who also have resistance to at least one fluoroquinolone are classified as having pre-extensively drug-resistant TB (Pre-XDR-TB). Pre-XDR-TB is MDR/RR-TB with additional resistance to at least one fluoroquinolone. In contrast, XDR-TB is Pre-XDR-TB with additional resistance to at least one Group A drug (bedaquiline or linezolid). Apart from these, MDR-TB remains a major public health concern and imposes a substantial burden on healthcare systems. According to recent data, globally, an estimated 390,000 people developed MDR/RR-TB in 2024. This estimate was lower than that of the previous year; drug-resistant TB is a major challenge globally [4]. Despite advances in therapeutic strategies, the treatment outcomes of MDR-TB remain poor, with a global treatment success rate of only about 50%, highlighting the need for more effective therapeutic interventions.

XDR-TB is difficult to treat, as it requires prolonged and more complicated therapy than MDR-TB. The treatment is associated with several disadvantages such as low efficacy, high cost, and toxicity. In 2018, WHO reported that the global treatment success rate for XDR-TB was 39% [5]. Bedaquiline is a newer anti-TB drug that has been incorporated into multidrug treatment regimens for drug-resistant TB [6,7]. Evidence from clinical trials and observational studies has demonstrated favorable treatment outcomes among patients receiving bedaquiline-containing regimens [7,8]. However, treatment outcomes in clinical practice may be influenced by the overall composition of the multidrug regimen, baseline disease characteristics, drug resistance patterns, treatment adherence, and other clinical factors [8]. Several observational and experimental studies have assessed the efficacy of bedaquiline against MDR-TB [9,10]. This study aimed to describe treatment outcomes and tolerability among patients with drug-resistant TB receiving a bedaquiline-containing multidrug regimen, with particular assessment of sputum conversion, laboratory parameters, adverse drug reactions (ADRs), and clinical response during the study follow-up period.

## Materials and methods

Research design

This was a hospital-based prospective observational cohort study conducted among patients with drug-resistant TB receiving a bedaquiline-containing multidrug regimen. The study was conducted at Zydus Medical College and Hospital, Dahod, from April 2024 to September 2025. A total of 64 patients with drug-resistant TB who attended the clinic were included according to predefined eligibility criteria. As the study had no comparator or control group, the observed treatment outcomes were interpreted as reflecting the overall treatment regimen rather than as evidence of bedaquiline's independent causal efficacy. The study included 64 patients with MDR-TB who attended the drug-resistant TB clinic. Both males and females were included in the study. Written and verbal informed consent was obtained from all participants. For children younger than 13 years, written informed consent was obtained from a parent or family member. Ethical approval was obtained from the Institutional Ethics Committee of Zydus Medical College and Hospital (approval number: ZMCH/IEC/018(06)-2024), and permission to conduct the study was obtained from the head of the department. Patients were selected according to predefined inclusion and exclusion criteria.

Inclusion and exclusion criteria

The study included patients aged >13 and <70 years with a confirmed diagnosis of TB. Eligible participants comprised individuals with microbiologically confirmed MDR-TB diagnosed using a cartridge-based nucleic acid amplification test, line probe assay, culture, or drug susceptibility testing. Only patients demonstrating resistance to both isoniazid and rifampicin were enrolled. Participants were required to have no documented history of significant cardiac disease, renal disease, or hepatic disorders.

Pregnant women were excluded from participation owing to the potential safety concerns associated with the study medication. Patients with a known history of hypersensitivity or allergic reaction to bedaquiline were also excluded from the study to minimize the risk of ADRs during treatment.

Procedure

Patients with MDR-TB who had attended the drug-resistant TB clinic at Zydus Medical College and Hospital were selected for the study based on predefined criteria. Bedaquiline was administered orally at a loading dose of 400 mg once daily for the initial 14 days, followed by a maintenance dose of 200 mg three times weekly on non-consecutive days. The duration of bedaquiline administration and the overall duration of the multidrug regimen were determined in accordance with the prevailing National Tuberculosis Elimination Programme (NTEP) treatment recommendations and individual patient eligibility. For patients receiving the six-month bedaquiline-pretomanid-linezolid-moxifloxacin regimen, bedaquiline was administered at 400 mg once daily during weeks 1-2, followed by 200 mg three times weekly from week 3 onwards. Pretomanid was administered orally at 200 mg once daily, linezolid at 600 mg once daily, and moxifloxacin at 400 mg once daily. In patients with documented fluoroquinolone resistance or in whom moxifloxacin could not be used, the regimen was modified in accordance with the prevailing NTEP recommendations and the patient's drug-susceptibility profile.

During the baseline visit, detailed demographic parameters, including age, gender, occupation, educational status, contact details, income, and area of residence, were collected and analyzed. The clinical parameters included complaints, diagnoses based on sputum analysis, both provisional and final chest X-ray results, personal, medical, and family history, general investigations, systemic examinations, laboratory analyses, including drug resistance patterns, and treatment details. These data were collected using a structured case report form and subsequently analyzed. All selected patients were followed monthly throughout the study period. Each follow-up visit included sputum analysis, chest X-ray, electrocardiogram (ECG), liver function tests (ALT, AST, alkaline phosphatase, and bilirubin), and serum electrolyte levels (calcium, magnesium, and potassium). Sputum specimens were collected at baseline and during scheduled follow-up visits and evaluated by sputum smear microscopy in accordance with the diagnostic and follow-up procedures used at the study center. Sputum conversion was assessed among patients with baseline sputum positivity. It was defined for this study as documentation of sputum smear negativity during follow-up after a previously positive baseline sputum examination. Patients were regularly monitored for the incidence of ADRs and the tolerability of the anti-TB treatment regimen. Clinical findings and laboratory results were recorded and analyzed at the follow-up visit. When longer or individualized bedaquiline-containing regimens were prescribed, the companion drugs were selected based on available drug-susceptibility testing results and program recommendations. Depending on individual eligibility and resistance patterns, companion drugs could include a fluoroquinolone such as levofloxacin or moxifloxacin, linezolid, clofazimine, cycloserine or terizidone, and other anti-TB drugs considered appropriate under the NTEP regimen. Details of the treatment regimen and any modification or discontinuation of individual drugs during follow-up were documented in the respective patient records.

Definition of primary outcome

The primary outcome of the study was sputum conversion during the follow-up period among patients receiving a bedaquiline-containing multidrug regimen. Sputum conversion was assessed using the diagnostic method routinely employed at the study center for the follow-up of patients with drug-resistant TB. Conversion was defined as achieving a negative sputum test result during follow-up, in accordance with the study center's established diagnostic and clinical procedures. Sputum status was assessed at the scheduled follow-up visits, and the proportion of patients demonstrating sputum conversion during the study period was recorded as the principal treatment outcome.

Statistical analysis

The study used Excel 2016 (Microsoft Corp., Redmond, WA, USA) for data management, and statistical analysis was performed in SPSS Statistics version 27 (IBM Corp., Released 2020; IBM SPSS Statistics for Windows, Version 27.0; Armonk, NY). Descriptive statistics were employed to summarize the demographic and clinical parameters of the study patients. Statistical tests were used to assess the significance of differences between groups, based on the nature of the data and their distribution.

Continuous variables were assessed for normality. Comparisons of categorical variables were performed using the chi-square test or Fisher's exact test, as appropriate. A two-sided p-value of <0.05 was considered statistically significant. Because multiple demographic, laboratory, and adverse-event comparisons were explored, the findings were interpreted cautiously as exploratory analyses. No causal inference regarding the independent effect of bedaquiline was made because of the observational design and absence of a comparator group.

## Results

Among 64 patients with MDR-TB, males constituted 40 (62.5%) and females 24 (37.5%). Sputum conversion was comparable between males (38, 95.0%) and females (23, 95.8%). Patients aged 15-24 years and 25-34 years each accounted for 15 (23.4%), with sputum conversion of 14 (93.3%) in both groups, while complete conversion was observed in the <15 years (2, 100.0%), 35-44 years (8, 100.0%), and 45-54 years (11, 100.0%) groups. Unemployed individuals comprised 25 (39.1%), and 24 (96.0%) achieved sputum conversion, whereas students showed the lowest conversion rate (3, 75.0%). Fever was the most common presenting complaint (49, 76.6%). Among patients weighing ≤40 kg, 32 (100.0%) achieved sputum conversion, while those weighing 51-60 kg had the lowest conversion rate (5, 83.3%). Diabetes mellitus (20, 31.2%) and hypertension (18, 28.1%) were the predominant comorbidities, with sputum conversion of 18 (90.0%) and 16 (88.9%), respectively. Smoking was the most frequent addiction (14, 21.9%), with complete sputum conversion (14, 100.0%). Overall, sputum conversion was achieved in 61 (95.3%) patients, with no statistically significant association between baseline characteristics and sputum conversion (all p > 0.05) (Table 1).

**Table 1:** Baseline and clinical parameters and their association with sputum conversion among patients with drug-resistant TB TB: tuberculosis, HIV: human immunodeficiency virus, COPD: chronic obstructive pulmonary disease

Variable	Category	Number of patients (n = 64)	Converted (n = 61)	Non-converted (n = 3)	p-value
Gender	Male	40 (62.5%)	38 (95.0%)	2 (5.0%)	–
	Female	24 (37.5%)	23 (95.8%)	1 (4.2%)	
Age group (years)	<15	2 (3.1%)	2 (100%)	0 (0%)	0.91
	15-24	15 (23.4%)	14 (93.3%)	1 (6.7%)	
	25-34	15 (23.4%)	14 (93.3%)	1 (6.7%)	
	35-44	8 (12.5%)	8 (100%)	0 (0%)	
	45-54	11 (17.2%)	11 (100%)	0 (0%)	
	≥55	13 (20.3%)	12 (92.3%)	1 (7.7%)	
Occupation	Laborers	11 (17.2%)	11 (100%)	0 (0%)	0.26
	Job	9 (14.1%)	9 (100%)	0 (0%)	
	Farmers	8 (12.5%)	8 (100%)	0 (0%)	
	Business	7 (10.9%)	6 (85.7%)	1 (14.3%)	
	Students	4 (6.2%)	3 (75.0%)	1 (25.0%)	
	Unemployed	25 (39.1%)	24 (96.0%)	1 (4.0%)	
Presenting complaint	Fever	49 (76.6%)	–	–	–
	Weight loss	34 (53.1%)	–	–	
	Cough with expectoration	29 (45.3%)	–	–	
	Weakness	28 (43.8%)	–	–	
	Cough	10 (15.6%)	–	–	
	Fever with chills	2 (3.1%)	–	–	
Body weight (kg)	≤40	32 (50.0%)	32 (100%)	0 (0%)	0.14
	41-50	25 (39.1%)	23 (92.0%)	2 (8.0%)	
	51-60	6 (9.4%)	5 (83.3%)	1 (16.7%)	
	≥60	1 (1.6%)	1 (100%)	0 (0%)	
Comorbidity	Diabetes mellitus	20 (31.2%)	18 (90.0%)	2 (10.0%)	0.99
	Hypertension	18 (28.1%)	16 (88.9%)	2 (11.1%)	
	Hypothyroidism	2 (3.1%)	2 (100%)	0 (0%)	
	HIV	2 (3.1%)	2 (100%)	0 (0%)	
	Sickle cell anemia	1 (1.6%)	1 (100%)	0 (0%)	
	Asthma	1 (1.6%)	1 (100%)	0 (0%)	
	COPD	1 (1.6%)	1 (100%)	0 (0%)	
Addiction	Smoking	14 (21.9%)	14 (100%)	0 (0%)	0.27
	Alcohol	9 (14.1%)	8 (88.9%)	1 (11.1%)	
	Tobacco	9 (14.1%)	9 (100%)	0 (0%)	

Among the anti-TB drugs evaluated, all 64 (100.0%) patients were resistant to rifampicin, and susceptibility to the other drugs was assessed. High resistance was also observed for KatG isoniazid (46, 71.7%) and InhA isoniazid (45, 70.3%), whereas susceptibility was observed in 18 (28.1%) and 19 (29.7%) patients, respectively. Levofloxacin resistance was identified in 34 (53.1%) patients, marginally exceeding the number of susceptible cases (30, 46.9%). In comparison, moxifloxacin demonstrated a more favorable susceptibility profile, with susceptibility observed in 41 (64.1%) patients and resistance in 23 (35.9%) patients. Injectable anti-TB drugs showed the highest susceptibility, with susceptibility observed in 63 (98.4%) patients remaining sensitive and only one (1.6%) patient exhibiting resistance. Overall, resistance was greatest to rifampicin and isoniazid, intermediate to levofloxacin, lower to moxifloxacin, and minimal to injectable drugs (Table 2). However, these results describe in vitro susceptibility patterns and do not establish comparative clinical efficacy among anti-TB drugs.

**Table 2:** Drug resistance patterns for anti-TB drugs among patients with drug-resistant TB TB: tuberculosis

Sr. No.	Anti-TB drug	No resistance-associated mutation detected	Resistance-associated mutation detected
1	Rifampicin	0 (0%)	64 (100%)
2	KatG isoniazid resistance marker	18 (28.1%)	46 (71.7%)
3	InhA isoniazid resistance marker	19 (29.7%)	45 (70.3%)
4	Levofloxacin	30 (46.9%)	34 (53.1%)
5	Moxifloxacin	41 (64.1%)	23 (35.9%)
6	Injectable drugs	63 (98.4%)	1 (1.6%)

Because rifampicin resistance was a predefined eligibility criterion, it was observed in all 64 enrolled patients, as expected. Therefore, this finding represents a baseline diagnostic characteristic of the study population rather than an independent study outcome. The drug-resistance profile was subsequently described in relation to additional anti-TB drugs and molecular markers of resistance. All 64 patients included in the study had been diagnosed with drug-resistant TB according to the diagnostic criteria and treatment records available at enrollment. Molecular drug-susceptibility testing demonstrated rifampicin resistance in all patients. The results for KatG and InhA represent mutation-specific molecular markers associated with isoniazid resistance and should not be interpreted independently as overall phenotypic susceptibility or resistance to isoniazid. Thus, the absence of resistance at one molecular target does not necessarily indicate isoniazid susceptibility, as resistance may be mediated by alterations at another target or by a different mechanism. Accordingly, the proportions reported as sensitive for KatG (28.1%) and InhA (29.7%) indicate the absence of resistance-associated mutations at those respective targets rather than the presence of rifampicin-monoresistant TB.

The laboratory parameters demonstrated variable changes across the three visits. Hemoglobin increased from 10.9 ± 1.9 g/dL at baseline to 11.1 ± 1.6 g/dL at the third visit, although the difference was not statistically significant (p = 0.084). Serum glutamic pyruvic transaminase (SGPT) increased substantially from 21.7 ± 8.6 to 52.5 ± 117.9 U/L, reaching borderline significance (p = 0.05), whereas serum glutamic-oxaloacetic transaminase (SGOT) remained essentially unchanged (p = 0.42). Total bilirubin increased from 0.8 ± 0.4 to 1.2 ± 0.5 mg/dL (p < 0.01). Albumin remained stable at 3.3 g/dL but showed a statistically significant change (p = 0.02). Sodium increased from 142.6 ± 6.3 to 145.3 ± 6.9 mEq/L (p < 0.01), while potassium remained stable despite statistical significance (p = 0.001). Urea, creatinine, and globulin showed no significant changes (Table 3).

**Table 3:** Comparison of laboratory parameters at baseline and the second and third visits among patients with drug-resistant TB TB: tuberculosis, SD: standard deviation, SGPT: serum glutamic pyruvic transaminase, SGOT: serum glutamic-oxaloacetic transaminase

Sr. No.	Parameter	1st visit (mean ± SD)	2nd visit (mean ± SD)	3rd visit (mean ± SD)	p-value (1st visit vs 3rd visit)
1	Hemoglobin (g/dL)	10.9 ± 1.9	11.1 ± 1.6	11.1 ± 1.6	0.084
2	SGPT (U/L)	21.7 ± 8.6	46.9 ± 96.1	52.5 ± 117.9	0.05
3	SGOT (U/L)	25.8 ± 15.8	25.7 ± 15.6	25.7 ± 15.3	0.42
4	Total bilirubin (mg/dL)	0.8 ± 0.4	1.1 ± 0.5	1.2 ± 0.5	<0.01
5	Urea (mg/dL)	19.9 ± 0.9	19.8 ± 6.6	19.9 ± 6.4	0.77
6	Creatinine (mg/dL)	1.5 ± 0.9	1.5 ± 0.9	1.5 ± 0.9	0.33
7	Albumin (g/dL)	3.3 ± 0.5	3.3 ± 0.4	3.3 ± 0.5	0.02
8	Globulin (g/dL)	3.6 ± 0.5	3.6 ± 0.5	3.6 ± 0.4	0.43
9	Sodium (mEq/L)	142.6 ± 6.3	145.5 ± 7.1	145.3 ± 6.9	<0.01
10	Potassium (mEq/L)	4.1 ± 0.5	4.1 ± 0.5	4.1 ± 0.5	0.001

During the second follow-up visit, abnormal electrolytes were the most frequently reported ADR, affecting 15 (23.4%) patients, followed by nausea in 13 (20.3%). Elevated SGPT/SGOT and abdominal pain were observed in four (6.3%) patients each, while headache occurred in two (3.1%) patients. Vomiting, arthralgia, and QT prolongation were the least common ADRs, each affecting one patient (1.6%). At the third follow-up visit, nausea and abnormal electrolytes declined to 12 (18.7%) each, remaining the most common ADRs. Abdominal pain increased slightly from four (6.3%) to five (7.8%), whereas vomiting and QT prolongation increased from one (1.6%) to two (3.1%) patients each. Arthralgia (1, 1.6%), headache (2, 3.1%), and elevated SGPT/SGOT (4, 6.3%) remained unchanged across both visits. Overall, most ADRs were either stable or showed a slight decline over time, with only abdominal pain, vomiting, and QT prolongation demonstrating marginal increases (Table 4).

**Table 4:** Incidence of ADRs during the second and third follow-up visits among patients with drug-resistant TB TB: tuberculosis, SGPT: serum glutamic pyruvic transaminase, SGOT: serum glutamic-oxaloacetic transaminase, ADRs: adverse drug reactions

Sr. No.	ADR	2nd visit (n = 64)	3rd visit (n = 64)
1	Nausea	13 (20.3%)	12 (18.7%)
2	Abdominal pain	4 (6.3%)	5 (7.8%)
3	Vomiting	1 (1.6%)	2 (3.1%)
4	Arthralgia	1 (1.6%)	1 (1.6%)
5	Headache	2 (3.1%)	2 (3.1%)
6	Elevated SGPT/SGOT	4 (6.3%)	4 (6.3%)
7	Abnormal electrolytes	15 (23.4%)	12 (18.7%)
8	QT prolongation	1 (1.6%)	2 (3.1%)

## Discussion

In the present study, sputum conversion exceeded 90% across all patient subgroups irrespective of age, sex, occupation, body weight, comorbidities, or addiction status, indicating favorable treatment outcomes with the bedaquiline-containing regimen. The treatment was also well tolerated, with nausea occurring in 20.3% of patients and QT prolongation in up to 3.1% of patients. Another study by Diacon et al. found that bedaquiline was associated with faster sputum conversion, with a median of 78 days compared with 129 days in the placebo group, along with a higher rate of culture conversion at 24 weeks. No bedaquiline resistance developed, whereas fluoroquinolone resistance was observed in the placebo group. Bedaquiline demonstrated an acceptable safety profile, with nausea and slight QT prolongation being the adverse symptoms [11]. Our findings further demonstrated favorable treatment outcomes, with sustained sputum conversion, preserved renal function, only mild alterations in liver function parameters, and a low frequency of clinically significant ADRs throughout the follow-up period. Another systematic review of 29 trials, including 23,358 patients, reported a significant positive effect of bedaquiline in pulmonary MDR-TB. Bedaquiline was associated with high rates of sputum conversion and cure, lower rates of treatment failure, and lower all-cause mortality compared with its absence. The findings were consistent across randomized and non-randomized trials.

Nevertheless, the use of bedaquiline was related to a high risk of cardiotoxicity (prolonged QT interval) and adverse events at grades 3-5. Thus, bedaquiline is a significant therapeutic strategy with tolerable side effects [12]. The study assessed the effectiveness and safety of bedaquiline as an alternative to second-line injectable (SLI) agents for managing MDR-TB in South Africa. The study involved 330 patients, including 162 treated with bedaquiline and 168 controls matched with bedaquiline, out of which 70.6% were HIV-positive. Twelve months after the commencement of treatment, a significantly higher proportion of patients treated with bedaquiline had favorable outcomes, demonstrating superiority over the control group that continued to receive SLI-based treatment. This was the result of higher rates of sustained sputum conversion and a lower rate of culture reversion (0.8% vs 10.3%).

Furthermore, the use of bedaquiline resulted in earlier culture conversion, while the mortality and loss-to-follow-up rates were similar in the two groups. The delay in initiating bedaquiline significantly increased the risk of failure to achieve sustained culture conversion. Additionally, hearing loss was the primary reason for discontinuation of SLI agent use [13]. Similarly, the present study observed a sputum conversion rate exceeding 90% and favorable treatment tolerance, consistent with favorable outcomes reported with bedaquiline-based therapy in routine clinical practice.

Another meta-analysis evaluated the management of drug-resistant TB, especially for MDR-TB and isoniazid-resistant TB. In a meta-analysis of 12,030 patients across 50 randomized controlled trials from 25 countries, the findings supported recommendations for constructing all-oral MDR-TB regimens, with reduced use of injectables due to their toxic effects. The study addressed the choice and number of drugs, treatment duration, the intensive and continuation phases, and surgery [14]. The observed universal rifampicin resistance (100%), high isoniazid resistance (>70%), preserved susceptibility to injectable drugs (98.4%), and higher susceptibility to moxifloxacin (64.1%) in our cohort further emphasize the importance of individualized all-oral regimens incorporating newer anti-TB agents such as bedaquiline. In another prospective multicenter Indian cohort of 620 MDR-TB patients on a bedaquiline-based regimen, 513/620 (83%) had culture conversion within six months, with a median conversion duration of 60 days.

The study group consisted of 354 (57%) with MDR-TB and fluoroquinolone resistance, 31 (5%) with SLI resistance, and 101 (16%) with XDR-TB. There were 100 patients (16.3%) with a QTc interval prolongation of ≥60 ms, demonstrating the significance of ECG monitoring. Seventy-three (12%) died; however, 56% of deaths occurred within the first six months, and according to the researchers, the bedaquiline-based regimens were effective with an improved tolerability profile [15].

Overall, the findings of the present study are largely consistent with the existing evidence supporting the efficacy and safety of bedaquiline in the management of drug-resistant TB. The high sputum conversion rate, favorable tolerability profile, preserved renal function, and only mild biochemical alterations observed during follow-up support the use of bedaquiline-containing regimens as a therapeutic option for patients with MDR-TB.

A major limitation of the present study is its single-center design, which may limit the generalizability of the findings to broader populations. Additionally, the absence of a comparator or control group and the relatively short follow-up period restricted the evaluation of the long-term efficacy, safety, and treatment outcomes associated with bedaquiline-based therapy.

## Conclusions

The study observed favorable short-term treatment outcomes and tolerability among patients receiving a bedaquiline-containing regimen. The present study showed a high sputum conversion rate among patients with drug-resistant TB, with no significant association between baseline demographic or clinical characteristics and treatment response. Drug susceptibility testing revealed universal resistance to rifampicin and high resistance to isoniazid and levofloxacin, whereas moxifloxacin and injectable anti-tubercular drugs showed comparatively greater susceptibility. Serial laboratory monitoring indicated that anti-TB therapy was generally well tolerated, with preserved renal function throughout follow-up and only mild but statistically significant alterations in selected liver function and electrolyte parameters. ADRs were predominantly mild, with nausea and electrolyte abnormalities being the most frequent events, while serious reactions such as QT prolongation occurred infrequently. However, the study did not include a comparator group, and bedaquiline was administered in combination with other anti-TB drugs; the independent contribution of bedaquiline to sputum conversion and other observed treatment outcomes cannot be determined. Furthermore, the limited follow-up duration does not permit conclusions regarding sustained treatment success, relapse, mortality, or long-term safety. The findings therefore provide observational evidence of short-term treatment outcomes and tolerability among patients receiving a bedaquiline-containing regimen, rather than definitive evidence of bedaquiline's independent efficacy or long-term safety.

Overall, the findings suggest that appropriate drug selection based on resistance patterns, together with regular clinical, laboratory, and adverse event monitoring, can achieve favorable treatment outcomes while maintaining an acceptable safety profile in patients with drug-resistant TB.
